# Evaluation of Lipid Accumulation Using Electrical Impedance Measurement under Three-Dimensional Culture Condition

**DOI:** 10.3390/mi10070455

**Published:** 2019-07-06

**Authors:** Daiki Zemmyo, Shogo Miyata

**Affiliations:** 1 Graduate School of Science and Technology, Keio University, 3-14-1 Hiyoshi, Yokohama 223-8522, Japan; 2 Department of Mechanical Engineering, Faculty of Science and Technology, Keio University, 3-14-1 Hiyoshi, Yokohama 223-8522, Japan

**Keywords:** electrical impedance measurement, three-dimensional cell culture, adipocyte, lipid droplet, 3T3-L1

## Abstract

The degeneration of adipocyte has been reported to cause obesity, metabolic syndrome, and other diseases. To treat these diseases, an effective *in vitro* evaluation and drug-screening system for adipocyte culture is required. The objective of this study is to establish an *in vitro* three-dimensional cell culture system to enable the monitoring of lipid accumulation by measuring electrical impedance, and to determine the relationship between the impedance and lipid accumulation of adipocytes cultured three dimensionally. Consequently, pre-adipocytes, 3T3-L1 cells, were cultured and differentiated to the adipocytes in our culture system, and the electrical impedance of the three-dimensional adipocyte culture at a high frequency was related to the lipid accumulation of the adipocytes. In conclusion, the lipid accumulation of adipocytes could be evaluated in real time by monitoring the electrical impedance during *in vitro* culture.

## 1. Introduction

Lipids are a critical factor for maintaining cellular energy homeostasis. Energy is stored as tryglycerides in lipid droplets when additional energy is ingested and hydrolyzed into fatty acids during energy shortage [1]. Although lipids contribute to the survival of living organisms, they cause diseases such as obesity and metabolic syndrome, both of which are related to lipid accumulation. Obesity, which is defined as an increase in adipose tissue, is a major problem worldwide, and considered to be caused by the intake of high calorie foods. Lysosomal diseases (LDs) are a type of genetic disease that cause metabolic disorders and are characterized by the accumulation of byproducts in the lysosome, owing to a defective catabolism. LDs are designated as an orphan disease [2] and occur in 1 out of 8000 live births [3]; therefore, a therapeutic approach is required to treat LDs. To establish medical treatment for various diseases related to metabolism effectively, it is essential to establish *in vitro* evaluation systems for lipid accumulation.

In adipocyte activity research, many types of assays have been used to evaluate the processes of adipogenesis and lipid accumulation. For example, Oil Red O staining is a primary evaluation method for the accumulation of lipid droplets in adipocytes [4,5]. Furthermore, glycerol-3-phosphate dehydrogenase activity [6], triglyceride content, and marker of genes such as PPARγ have been measured to evaluate adipocyte metabolism. However, these assays are only end-point assays and do not enable the real-time monitoring of adipocyte activities. A histological assessment is required to obtain photomicrographs such that they can be analyzed to evaluate the degree of stained area. Therefore, it is essential to develop a new technique to evaluate the degree of lipid accumulation quantitatively and conveniently in real time.

Electric cell-substrate impedance sensing was established by Giaever and Keese in 1984 [7]. It is a technique for measuring cellular properties, such as cell density [8], cell aging [9], cell adhesion [10], and cytotoxicity [11]. Furthermore, this sensing technology has been applied in monitoring cell differentiation, for example, stem cell differentiation [12] and neural differentiation [13]. It can also be applied in cell monitoring without cellular damage and hence enable a real-time evaluation. Because adipose tissue exhibits higher electrical impedance than other tissues, it is considered that the electrical characteristics of adipocytes would change during lipid accumulation. Several reports have been published regarding the monitoring of electrical characteristics during lipid accumulation in adipocytes [14,15]. However, few studies have evaluated the relationship between the electrical characteristics and the amount of lipid droplets in adipocytes. Furthermore, the evaluation was not performed under physiological conditions, because the measurement was only performed in the two-dimensional culture of adipocytes. Therefore, a platform for a screening system or a fundamental study to evaluate lipid accumulation under a three-dimensional culture is required.

In this study, we developed a cell culture device that can simultaneously measure electrical characteristics under a three-dimensional cell culture condition. Moreover, the relationship between the electrical characteristics and the lipid accumulation of adipocytes cultured three-dimensionally was determined using our novel cell culture device.

## 2. Materials and Methods 

### 2.1. Impedance Measurement of Three-Dimensional Cell Culture

**Figure 1:**
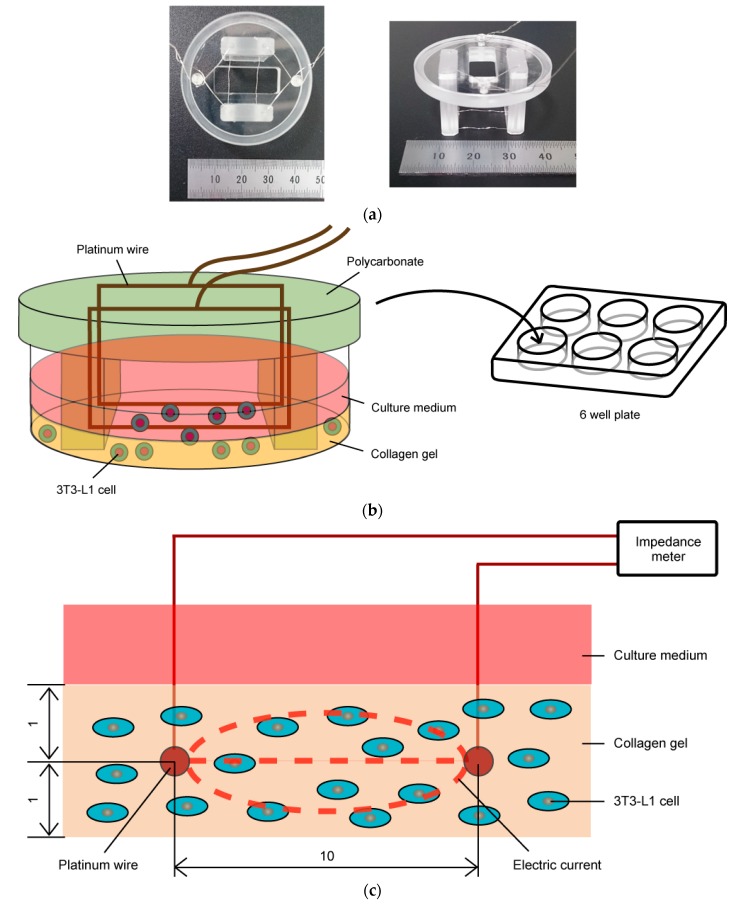
(**a**) Cell culture device for electrical impedance measurement. (**b**) Two platinum wires were fixed with a polycarbonate fixture and placed inside the 3T3-L1-cell-embedded collagen gel. It was placed within a 6-well cell-culture plate. (**c**) Cross-sectional view of the culture region of impedance measurement device. Voltage was applied between the two platinum wires.

An impedance measurement device was developed to evaluate the electrical characteristics of adipocytes cultured under three-dimensional conditions (Figure 1). The device was composed of two platinum wires bridged between two polycarbonate fixtures in each well of a six-well cell culture plate. A cell-seeded collagen gel disk could be cultured in each well. The two platinum wires were embedded in the gel and held by two polycarbonate fixtures to be positioned in parallel with each other (10 mm apart) and 1 mm height from the bottom surface of the six-well plate. The length of each platinum wire in the cell-seeded gel was set as 20 mm, and the diameter was 0.2 mm. Each platinum wire passed through the inside of the polycarbonate fixture and was connected to the electrical impedance meter (Chemical Impedance Meter, Hioki, Japan). The complex impedance between two platinum wires was measured to evaluate the electrical characteristics of the three-dimensionally cultured adipocytes. Furthermore, this device could be set in a CO_2_ gas incubator to enable real-time impedance monitoring during cell culture.

**Figure 2:**
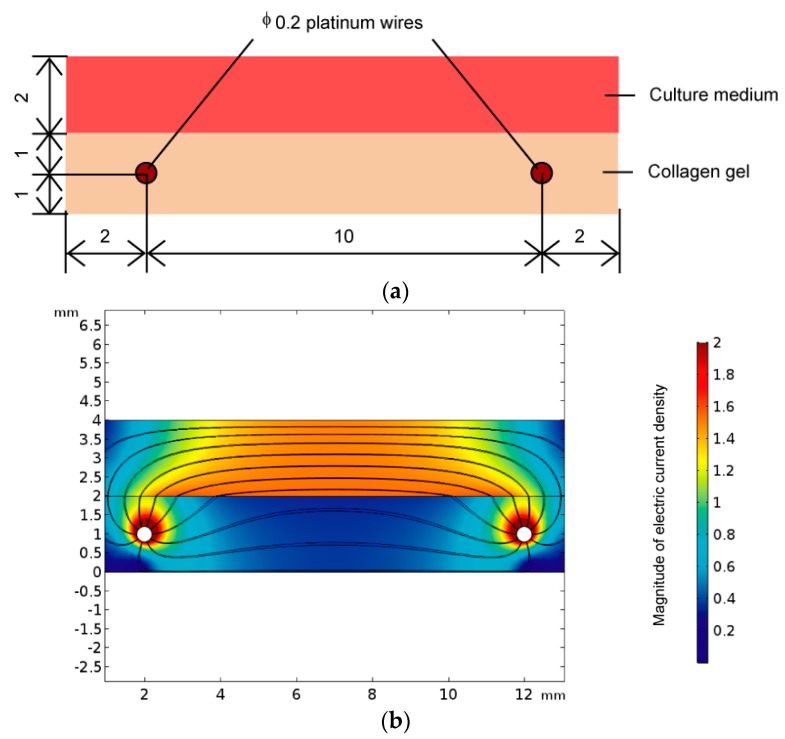
(**a**) Numerical analysis model of electrical field. (**b**) Pathway of electric currents between two platinum wires in the cell culture region. Streamlines show the pathway of the electric current, and the contour plot shows the magnitude of the electric current density.

To evaluate how to pass electric currents between the two platinum wires, numerical analysis was performed to evaluate the electric field in the cultured area of the device, using finite element analysis software (COMSOL Multiphysics Version 5.2a, COMSOL Inc., Stockholm, Sweden). In the analysis, the conductivity and relative permittivity were set as follows: 1.38 S/m [16,17,18] and 80 [16] in culture medium; 0.30 S/m [19] and 2.28 [20] in collagen gel. The result of this numerical analysis indicated that electric currents passed through both areas of culture medium and collagen gel (Figure 2). The proportion of electric current that flowed in the collagen gel was approximately 20% of the total electric current between the two wires and was considered as sufficient to reflect the electrical characteristics of the cell-seeded collagen gel.

### 2.2. Evaluation of Characteristics of Living Cell

Intracellular and extracellular properties can be evaluated separately by measuring the electrical characteristics of cells in multiple frequency bands. Ions in a cytoplasm are gathered around the surface of a cell membrane to increase the apparent dielectric constant of cytoplasm if the cells are subjected to a low-frequency alternating-current (AC) electric field (Figure 2). The electric currents can pass through the surface of the cell membrane but not through the cytoplasm because of the increased dielectric constant of the cytoplasm. By contrast, if cells are subjected to a high-frequency AC electric field, the ions inside the cells cannot follow the change in electric field to decrease the dielectric constant of the cytoplasm. Furthermore, the apparent electrical impedance decreases in relation to the decrease in dielectric properties of the cytoplasm to enable the penetration of electric currents into the cell membrane. Therefore, if a high-frequency AC electric field is applied to living cells, the measured data would reflect the electrical characteristics of intracellular constituents.

As for the two-dimensional culture of pre-adipocytes (3T3-L1 cells) and adipocytes, it was reported that the effect of lipid accumulation on the change in impedance at lower frequencies (10–65 kHz) was negligible [15]. Meanwhile, the impedance at higher frequencies (1–15 MHz) changed according to the lipid accumulation [21]. These results indicate that lipid accumulation could not affect the impedance at lower frequencies (approximately 10 kHz) but could affect the impedance at higher frequencies (1 MHz). The impedance at lower frequencies could be related partially to the electrical characteristic of cell membranes and primarily to the experimental setup of the measurement device. Therefore, we propose an impedance at a higher frequency corrected by a lower frequency, i.e., Z(1 MHz)’ = Z(1 MHz) − Z(10 kHz) as an evaluation parameter for the change in intracellular constituents, especially for the lipid accumulation.

### 2.3. Cell Culture with Impedance Measurement

Murine pre-adipocyte 3T3-L1 cells were maintained in 75 cm^2^ flasks in Dulbecco’s modified eagle medium (DMEM, high glucose, Gibco), supplemented with 10% Equa-FETAL (Equa FETAL, EF-0500-A, Atlas Biologicals, Fort Collins, CO, USA) and 2% antibiotic–antimycotic (Antibiotic-Antimycotic Mixed Stock Solution, Nacalai Tesque, Japan) in a humidified CO_2_ incubator (5% CO_2_ at 37 °C). From a cryopreserved stock, 3T3-L1 cells were passaged twice before being embedded in collagen gel. In this study, two types of basal mediums were used for 3T3-L1 cells to evaluate the effect of glucose concentration on lipid accumulation. The lipid metabolism is closely related to the glucose metabolism in adipocyte. DMEM with high glucose (4.5 g/L) was used for the high-glucose condition (high-G group), and DMEM with low glucose (1.0 g/L) was used for the low-glucose condition (low-G group). The basal medium supplemented with 1.0 μM dexamethasone, 0.50 mM isobutyl-methylxanthine, and 10 μg/mL insulin was prepared as an adipocyte differentiation medium. A basal medium with 10 μg/mL insulin was prepared for the maintenance culture after adipocyte differentiation. A collagen gel without 3T3-L1 cells was prepared as a no-cell group to confirm the effect of 3T3-L1 cells on the electrical impedance.

**Figure 3:**
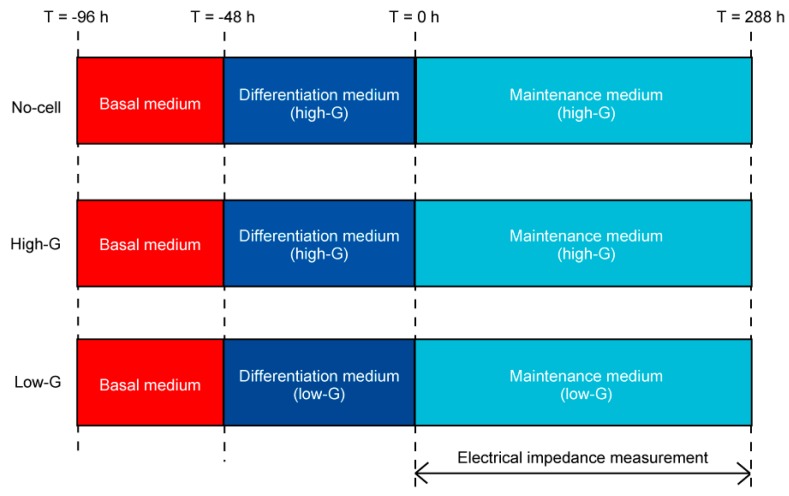
Time-course of cell culture experiments. The 3T3-L1 cells were cultured three-dimensionally in basal medium for 48 h following adipogenesis in differentiation medium for 48 h. After adipogenesis (t = 0 h), the differentiated 3T3-L1 cells were cultured in maintenance medium and the electrical impedance of the cells was measured until t = 288 h.

In the high-G and low-G groups, the 3T3-L1 cells were suspended with 2.4 mg/mL neutralized type-I collagen solution at a concentration of 1.0 × 10^6^ cells/mL. A 2.0 mL of cell-suspended collagen solution was poured into the well of the impedance measurement device and gelled for 30 min at 37 °C in the incubator. In the no-cell group, the neutralized type-I collagen solution without cells was poured into the well of the device, following gelation in the incubator. An amount of 2.0 mL of basal medium was poured into the well after the gelation, and the cell-seeded collagen gel was cultured for 48 h. Subsequently, the culture medium was changed to a differentiation medium to induce the adipogenesis of 3T3-L1 cells. Following adipogenesis for 48 h, the culture medium was changed to a maintenance medium. In this experiment, at the start of the maintenance culture, the time was set as t = 0 h (Figure 3). The 3T3-L1 cells in the collagen gel were cultured until t = 288 h. The culture medium was changed every 48 h during the maintenance culture. 

### 2.4. Biochemical Characterization

In this study, to focus on real-time and in situ monitoring of lipid accumulation, the evaluation of lipid accumulation was performed without fluorescent staining of cells. The lipid accumulation was evaluated from the phase-contrast images of the cells. The images were acquired at the center plane between the top and bottom surface of cell-seeded gel. In our preliminary studies, the lipids in 3T3-L1 cells were stained by Oil red O and demonstrated sphere-like shapes (Figure S1) [22]. Based on these results, the circle-like constituents from 1 μm to 10 μm in the 3T3-L1 cells were defined as lipids, and the total area of these constituents was measured for the evaluation of lipid accumulation.

To examine the effect of cell mass on electrical characteristics in collagen gel, the cell number was evaluated by the quantification of total DNA amount in collagen gel. It was reported that the total DNA was related to the cell number in hydrogels or living tissues [23,24]. After the cell culture experiment, the samples were lyophilized overnight and treated with 125 μg/mL papain solution at 60 °C for 6 h to solubilize the collagen gel. The total DNA amount in the digested specimen was determined using a fluorescence spectrophotometer (Qubit 2.0 Fluorometer, Life Technologies, Carlsbad, CA, USA). 

### 2.5. Statistical Analysis

Most of the data are representative of three individual experiments with similar results. For each group, 4 samples (n = 4) were analyzed per time point, and each data point represents the mean and standard deviation. Data from each experimental group were examined for significant differences using t-tests. Pearson’s correlation analyses were also carried out on pooled data sets of high-G and low-G groups to determine the relationship between the electrical impedance and lipid accumulation.

## 3. Results and Discussion

### 3.1. Effect of Glucose Concentration on Proliferation and Lipid Accumulation of 3T3-L1 Cells

**Figure 4:**
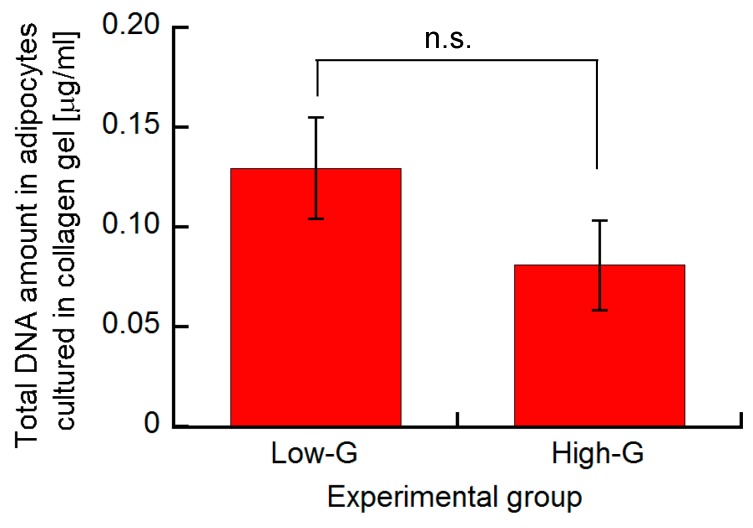
Total DNA amount in adipocytes cultured in collagen gel. Data are presented as mean ± S.D., n = 4.

In this study, to evaluate the number of living cells in collagen gel at the end of culture time, the amount of total DNA in the cultured specimen was quantified using a fluorometric assay. The quantification of total DNA was used for the evaluation of cell number in living tissues or three-dimensional cultures [25]. No significant differences were found between the DNA amount of high-G and low-G groups (Figure 4). Therefore, it was assumed that the change in electrical impedance was primarily dependent on the lipid accumulation and adipogenesis of 3T3-L1 cells. 

**Figure 5:**
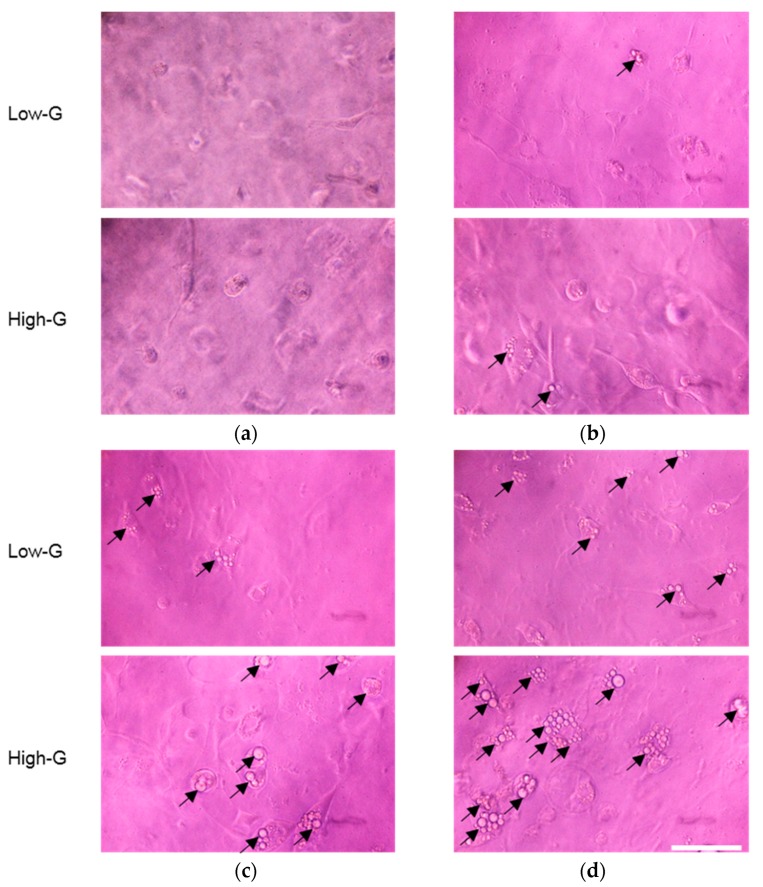
Phase-contrast images of 3T3-L1 cells cultured in collagen gel at (**a**) −48 h, (**b**) 96 h, (**c**) 192 h, and (**d**) 288 h post adipogenesis. Lipid droplets were accumulated after t = 96 h (black arrows indicate lipid droplets). Scale bar: 50 μm.

For the evaluation of lipid accumulation in 3T3-L1 cells, the phase-contrast images of the cells in the collagen gel were acquired every 48 h. Before adipocyte differentiation, at t = −48 h, the cells demonstrated similar morphology and the cell number was similar in both the high-G and low-G groups (Figure 5a). At t = 96 h, small lipid droplets were observed in the cells in both groups (Figure 5b); therefore, it was revealed that the 3T3-L1 cells in collagen gel differentiated into adipocytes and accumulated lipid droplets. With increasing culture time, the number of lipid droplets increased in both groups and the size of each droplet became larger (Figure 5c,d). Therefore, it was suggested that the 3T3-L1 pre-adipocytes could be cultured three-dimensionally and the amount of lipid droplets could increase with culture time.

**Figure 6:**
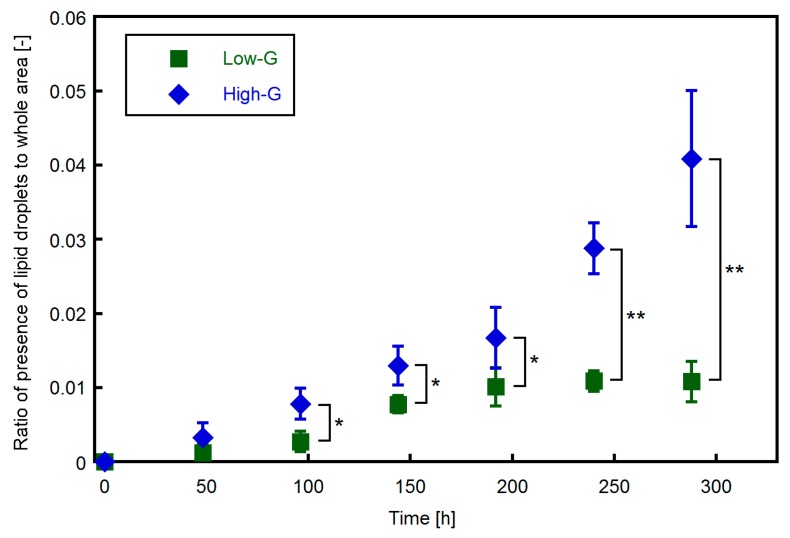
Area proportion of lipid droplets in phase-contrast microscopic images. The amount of lipid droplets in the high-G group was significantly larger than that in the low-G group after t = 96 h. Data are presented as mean ± S.D., n = 4. * and ** indicate significant differences between low-G and high-G groups (*: *p* < 0.05, **: *p* < 0.01).

The area ratio of lipid droplets to the entire imaged area at every 48 h is shown in Figure 6. During the adipocyte differentiation period (from t = −48 h to 0 h), no lipid droplets were detected in both the high-G and low-G groups, whereas a small number of lipids was observed at t = 48 h. In the low-G group, the area ratio of the lipid droplet increased at a rate of approximately 0.5% per 48 h until t = 192 h and did not change after t = 192 h. Meanwhile, in the high-G group, the area ratio increased at a rate of approximately 0.5% per 48 h until t = 192 h and at a rate of approximately 1% per 48 h after t = 192 h. At each time point, the proportion of lipid droplets in the high-G group was larger than that in the low-G group and a significant difference was observed after t = 96 h. Therefore, it was considered that the concentration of glucose in the culture medium would affect the accumulation of lipid droplets in adipocytes.

### 3.2. Electrical Impedance of 3T3-L1 Cells Cultured Three-Dimensionally

**Figure 7:**
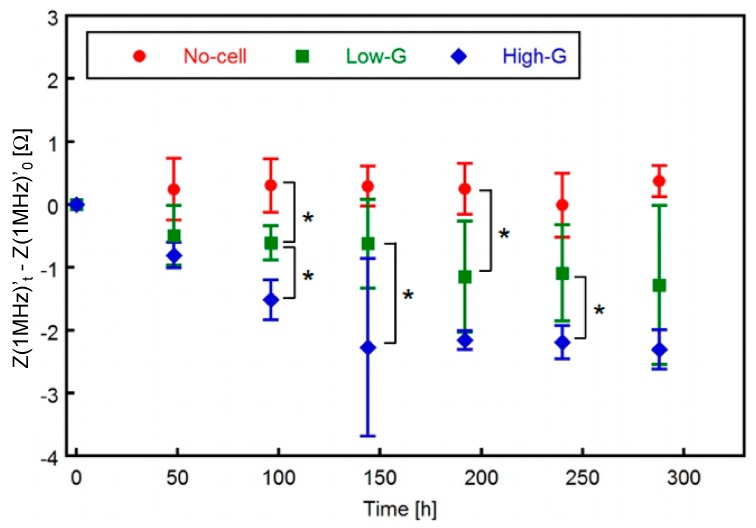
Change in Z(1 MHz)’_t_ − Z(1 MHz)’_0_ in three experimental groups. Both the values of low and high-G groups decreased with increase in culture time. Data are presented as mean ± S.D., n = 4. * indicates a significant difference between low-G and high-G groups, *p* < 0.05.

As described in the “Materials and Methods” section, the corrected impedance at higher frequencies, Z(1 MHz)’ = Z(1 MHz) − Z(10 kHz), was defined as the evaluation of the electrical impedance. The change in Z(1 MHz)’ from t = 0 h, Z(1 MHz)’_t_ − Z(1 MHz)’_0_, is shown in Figure 7. In the no-cell group, the Z(1 MHz)’ did not change during the culture time. Meanwhile, the Z_1MHz_’ of the low-G group decreased monotonically and that of the high-G group decreased until t = 144 h and saturated from t = 144 h to 288 h. 

**Figure 8:**
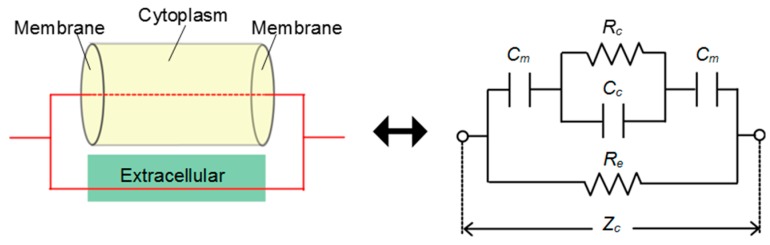
Equivalent circuit of a living cell. Cytoplasm was modeled as a parallel circuit of resistance R_c_ and capacitance C_c_, cell membrane as capacitance C_m_, and extracellular medium as resistance R_e_.

Significant differences between the Z(1 MHz)’_t_ − Z(1 MHz)’_0_ values of the no-cell and cell-seeded groups (high-G and low-G groups) were detected at t = 96 h and 192 h. Moreover, a significant difference between the values of low-G and high-G groups was also observed from 96 h to 144 h and 240 h. In previous studies, the electric property of a living cell was modeled as an equivalent circuit consisting of resistances and capacitance (Figure 8) [25]. At higher frequencies, the dielectric constant of the inner cell constituents would decrease with lipid accumulation, thus resulting in the decrease in conductance. Therefore, the impedance at higher frequencies was considered to decrease with lipid accumulation. Our experimental results were consistent with the analysis based on the equivalent circuit model of a cell. As mentioned in Section 3.1, no significant difference was found between the cell numbers of the high-G and low-G groups. The difference in the values of Z(1 MHz)’_t_ − Z(1 MHz)’_0_ could be related to lipid accumulation in the cell.

### 3.3. Relationship between Lipid Accumulation and Electrical Impedance

**Figure 9:**
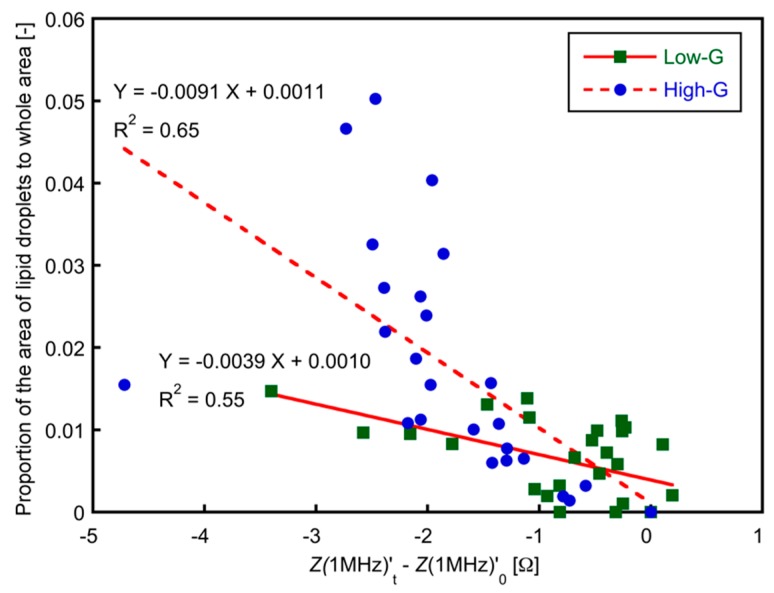
The relationship between Z(1 MHz)’_t_ − Z(1 MHz)’_0_ and the proportion of area of lipid droplets to entire area. The entire data in low-G and high-G groups are plotted. A significant negative correlation was found (R^2^ = 0.49, *p* < 0.05).

The relationship between the time change in electrical impedance at high frequencies, Z(1 MHz)’_t_ − Z(1 MHz)’_0_ and the area ratio of lipid droplets to the total area of phase-contrast images is shown in Figure 9. Significant negative correlations were found between the change in Z(1 MHz)’_t_ − Z(1 MHz)’_0_ and the area proportion of the lipid droplets (R^2^ = 0.55, *p* < 0.05 for low-G group; R^2^ = 0.65, *p* < 0.05 for high-G group). These experimental results were qualitatively consistent with the equivalent circuit model of a cell (Figure 8) [25]. It was suggested that the amount of lipid droplets in a three-dimensional adipocyte culture could be evaluated by monitoring the change in impedance at high frequencies, Z(1 MHz)’ during an *in vitro* culture.

## 4. Conclusions

In this study, a cell culture device that could simultaneously measure electrical characteristics under a three-dimensional culture was developed. The pre-adipocytes, i.e., 3T3-L1 cells, were cultured and differentiated in collagen gel and their electric property was evaluated during the culture. The relationship between the amount of lipid droplets and the electrical impedance of three-dimensionally cultured adipocytes was evaluated. Results indicated that the lipid accumulation of adipocytes could be evaluated in real time by measuring the impedance at higher frequencies.

## Supplementary Materials

The following are available online at https://www.mdpi.com/2072-666X/10/7/455/s1, Figure S1: (**a**) Phase-contrast and (**b**) Oil red O stained images of differentiated 3T3-L1 cells. Scale bar: 100 μm.
