# Supplementary material for: Evaluation of Lipid Accumulation Using Electrical Impedance Measurement under Three-Dimensional Culture Condition
Source: Micromachines (Basel). 2019 Jul 6;10(7):455. doi: 10.3390/mi10070455 (PMC6680657; doi:10.3390/mi10070455)
Supplement: Supplementary file 1 [file micromachines-10-00455-s001.pdf]

micromachines

Supplementary Materials: Evaluation of Lipid Accumulation Using Electrical Impedance Measurement under Three-Dimensional Culture Condition

Daiki Zemmyo [1] and Shogo Miyata [2,*]

1 Graduate School of Science and Technology, Keio University, 3-14-1 Hiyoshi, Yokohama 223-8522, Japan
2 Department of Mechanical Engineering, Faculty of Science and Technology, Keio University, 3-14-1 Hiyoshi, Yokohama 223-8522, Japan
* Correspondence: miyata@mech.keio.ac.jp; Tel.: +81-45-566-1827

3T3-L1 cells were differentiated to adipocyte and stained by Oil-red O. The circle-like constituents were stained as lipid droplets.

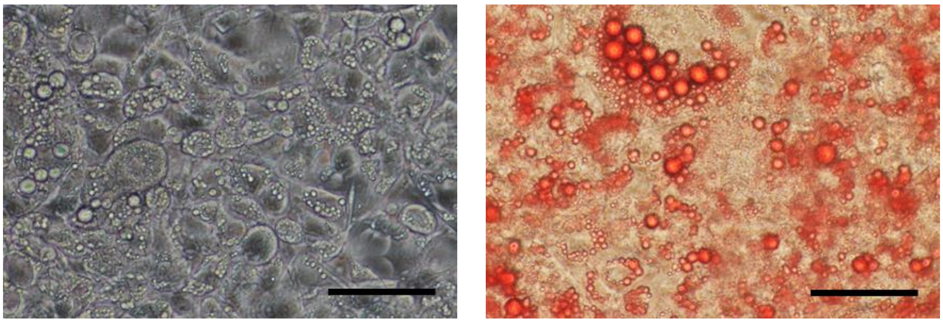

(**a**) (**b**)

Figure S1. (**a**) Phase-contrast and (**b**) Oil red O stained images of differentiated 3T3-L1 cells. Scale bar: 100 μm.
